# Cross-cultural data on romantic love and mate preferences from 117,293 participants across 175 countries

**DOI:** 10.1038/s41597-025-05365-2

**Published:** 2025-07-01

**Authors:** Marta Kowal, Piotr Sorokowski, Biljana Gjoneska, Katarzyna Pisanski, Gerit Pfuhl, Leonardo Aguilar, Steve M. J. Janssen, Benjamin Gelbart, Patrícia Arriaga, Jan Antfolk, Katarina Zvončáková, Linda H. Lidborg, Jorge Contreras-Garduño, Mikhail V. Kozlov, Taciano L. Milfont, Marco A. C. Varella, Valerija Križanić, Mahmoud Boussena, Tina Kavčič, Diana Ribeiro da Silva, Brahim Hamdaoui, Fatima Zahra Sahli, Karlijn Massar, Eliane Deschrijver, Tatsunori Ishii, Hakan Cetinkaya, Oksana Senyk, Farida Guemaz, Koen Ponnet, Yahya Don, Dušana Šakan, Gyesook Yoo, Ravit Nussinson, Joaquín Ungaretti, Ali R. Can, Izzet Duyar, Jiří Čeněk, Joao Carneiro, Norbert Meskó, Luca Kozma, Ellen K. Nyhus, Mona Vintila, Oulmann Zerhouni, Farid Pazhoohi, Maja Zupančič, Sinem Söylemez, Austin H.-E. Wang, Marietta Papadatou-Pastou, Irena Pavela Banai, Pavol Prokop, Mohd Sofian Omar Fauzee, Reza Afhami, Jean C. Natividade, Roberto Baiocco, Mara Morelli, Toivo Aavik, Ezgi Toplu-Demirtaş, Singha Tulyakul, Anna Wlodarczyk, Razieh Chegeni, Anabela C. Santos, Dmitry Grigoryev, Dmitrii Dubrov, Dimitri Chubinidze, Gözde Ikizer, Nana Burduli, Johanna Czamanski-Cohen, Rizwana Amin, Petros Roussos, Evgeniya Hristova, Rūta Sargautytė, Ekaterine Pirtskhalava, Tenuunjargal Avirmed, Arooj Najmussaqib, Abdelilah Charyate, Shagufta Batool, Tatiana Volkodav, Yoshihiko Kunisato, Yuki Yamada, Asako Toyama, Mariia Perun, Seda Dural, Tetyana Mandzyk, Anna Studzinska, Ognen Spasovski, Felipe E. García, Caterina Grano, Merve Boğa, Mehmet Koyuncu, Sangeeta Singh, Ju Hee Park, Derya Atamtürk, Samuel Lins, Martin Pírko, David Lacko, Balazs Aczel, Ferenc Kocsor, Ádám Putz, Tobias Otterbring, Pavol Kačmár, Efisio Manunta, Théo Besson, Nasim Ghahraman Moharrampour, Çağlar Solak, Bojana M. Dinić, Ignacio Estevan, Merve Topcu Bulut, Nicolas Kervyn, Moises Mebarak, Jackson G. Lu, Nejc Plohl, Bojan Musil, Adil Samekin, Kirill G. Miroshnik, Clément Cornec, Isabella Giammusso, Ulf-Dietrich Reips, Maria Rosa Miccoli, Miriam Parise, Sabrina Stöckli, Tiago Marot, Sibele D. Aquino, Amanda Londero-Santos, Antonio Chirumbolo, Aybegum Memisoglu-Sanli, Jaroslava V. Valentova, Cemre Karaarslan, Ivana Hromatko, Kevin Sevag Kertechian, Ogeday Çoker, Matheus F. Ribeiro, Carlota Batres, Ilker Dalgar, Stephanie J. Eder, Katarina Mišetić, Marios Argyrides, Vita Mikuličiūtė, Silvia Mari, Elisabeth Oberzaucher, Kathrin Masuch, Alan D. A. Mattiassi, Salma S. Omar, Elena Piccinelli, Eda Ermagan Caglar, Diogo Lamela, David A. Frederick, Aleksander Kobylarek, Ma Criselda T. Pacquing, Marc Eric S. Reyes, Marcos Zumárraga-Espinosa, Feten Fekih-Romdhane, Talía Gómez Yepes, Edgardo Etchezahar, Katarzyna Galasinska, Jan P. Röer, Ayşegül Şahin, Miguel Landa-Blanco, Izuchukwu L. G. Ndukaihe, Arkadiusz Urbanek, Chee-Seng Tan, Rita Castro, Ksenija Cunichina, Anna Krasnodębska, Daniel Conroy-Beam, Franciszek Ostaszewski, Izabela Chałatkiewicz, Beatriz Abad-Villaverde, Bastien Trémolière, Alexios Arvanitis, Gulmira T. Topanova, William J. Chopik, Grace Akello, Ariela F. Pagani, Silvia Donato, Peter Fedor, Tomasz Frackowiak, Simon Ozer, Marlon Mayorga-Lascano, Farah Khan, Maryanne L. Fisher, Princess Lovella G. Maturan, Tatiana Semenovskikh, Sanjana Dutt, William Tamayo-Agudelo, Gulnara Ismukhanova, Laith Al-Shawaf, Luisa Angelucci, Adam Bode, Sercan Balım, Jovi C. Dacanay, Chiemezie S. Atama, Kai A. D. Morgan Campbell, Tchilissila A. Simões, Barış Özener, Paula Błauciak, Filipe Prazeres

**Affiliations:** 1 IDN Being Human Lab - Institute of Psychology, University of Wrocław, Wrocław, Poland; 2 Institute of Psychology, University of Wrocław, Wrocław, Poland; 3 Macedonian Academy of Sciences and Arts, Skopje, North Macedonia; 4 CNRS French National Centre for Scientific Research, DDL Dynamics of Language Laboratory, University of Lyon 2, Lyon, France; 5 ENES Bioacoustics Research Lab, Jean Monnet University of Saint-Etienne, Saint-Etienne, France; 6 Department of Psychology, Norwegian University of Science and Technology, Trondheim, Norway; 7 School of Psychology, Central University of Venezuela, Caracas, Venezuela; 8 School of Psychology, University of Nottingham Malaysia, Semenyih, Malaysia; 9 Department of Psychological and Brain Sciences, University of California, Santa Barbara, Santa Barbara USA; 10 Departament of Psychology & CIS-Iscte, ISCTE - Instituto Universitário de Lisboa, Lisbon, Portugal; 11 Faculty of Arts, Psychology and Theology, Åbo Akademi University, Turku, Finland; 12 Institute of Psychology, Czech Academy of Sciences, Brno, Czech Republic; 13 Department of Psychology, Masaryk University, Brno, Czech Republic; 14 Department of Psychology, Durham University, Durham, United Kingdom; 15 Escuela Nacional de Estudios Superiores Unidad Morelia, Universidad Nacional Autónoma de México, Morelia, México; 16 Department of Biology, University of Turku, Turku, Finland; 17 School of Psychological and Social Sciences, University of Waikato, Tauranga, New Zealand; 18 Department of Experimental Psychology, Institute of Psychology, University of São Paulo, São Paulo, Brazil; 19 Department of Psychology, Faculty of Humanities and Social Sciences, J. J. Strossmayer University of Osijek, Osijek, Croatia; 20 Department of Psychology, University Mohamed lamine Debaghine Setif2, Setif, Algeria; 21 Department of Psychology, Faculty of Arts, University of Ljubljana, Ljubljana, Slovenia; 22 Faculty of Health Sciences, University of Ljubljana, Ljubljana, Slovenia; 23 CINEICC – Center for Research in Neuropsychology and Cognitive Behavioral Intervention, University of Coimbra, Coimbra, Portugal; 24 Departement of Sociology, Ibn Tofail University, Kénitra, Morocco; 25 Ibn Tofail University, Kenitra, Morocco; 26 Department of Work & Social Psychology, Maastricht University, Maastricht, The Netherlands; 27 School of Psychology, University of Sydney, Sydney, Australia; 28 Department of Psychology, Japan Women’s University, Tokyo, Japan; 29 Department of Psychology, Yaşar University, Izmir, Turkey; 30 Department of Humanities and Social Sciences, WSB Merito University in Gdansk, Gdansk, Poland; 31 Department of Psychology and Eduactional sciences and Orthophony, Mohamed Lamine Debaghine University Setif2, Setif, Algeria; 32 Faculty of Social Sciences, Ghent University, Ghent, Belgium; 33 School of Education (SoE), Universiti Utara Malaysia, Alor Setar, Malaysia; 34 Department of Psychology, Faculty of Legal and Business Studies dr Lazar Vrkatić, Novi Sad, Serbia; 35 Department of Child & Family Studies, Kyung Hee University, Seoul, Republic of Korea; 36 Department of Education and Psychology, The Open University of Israel, Raanana, Israel; 37 Institute for Information Processing and Decision Making, University of Haifa, Haifa, Israel; 38 Faculty of Education, Valencian Internacional University, Valencia, Spain; 39 Department of Anthropology, Hatay Mustafa Kemal University, Hatay, Turkey; 40 Department of Anthropology, Istanbul University, Istanbul, Turkey; 41 Department of Social Studies, Mendel University in Brno, Brno, Czechia; 42 Interdisciplinary Research Team on Internet and Society, Masaryk University, Brno, Czechia; 43 Department of Psychology, University of Porto, Porto, Portugal; 44 Institute of Psychology, University of Pécs, Pécs, Hungary; 45 Division of Psychology, University of the West of Scotland, Paisley, United Kingdom; 46 Department of management, University of Agder, Kristiansand, Norway; 47 Psychology Department, West University of Timisoara, Timisoara, Romania; 48 Centre de Recherche sur les Fonctionnements et Dysfonctionnements Psychologiques, Université de Rouen Normandie, Rouen, France; 49 Laboratoire Parisien de Psychologie Sociale, Université Paris Nanterre, Nanterre, France; 50 School of Psychology, University of Plymouth, Plymouth, United Kingdom; 51 Department of Psychology, Manisa Celal Bayar University, Manisa, Turkey; 52 Department of Political Science, University of Nevada, Las Vegas, Las Vegas, USA; 53 Department of Primary Education, National and Kapodistrian University of Athens, Athens, Greece; 54 Department of Environmental Ecology, Comenius University, Bratislava, Slovakia; 55 Department of Animal Ecology, Comenius University, Bratislava, Slovakia; 56 Faculty of Education and Liberal Arts, INTI International University, Nilau, Malaysia; 57 Department of Art Studies, Tarbiat Modares University, Tehran, Iran; 58 Department of Psychology, Pontifical Catholic University of Rio de Janeiro, Rio de Janeiro, Brazil; 59 Department of Developmental and Social Psychology, Sapienza University of Rome, Rome, Italy; 60 Department of Dynamic and Clinical Psychology, and Health Studies, Sapienza University of Rome, Rome, Italy; 61 Institute of Psychology, University of Tartu, Tartu, Estonia; 62 Department of Psychological Counseling and Guidance, MEF University, İstanbul, Turkey; 63 Faculty of Education, Thaksin University, Songkhla, Thailand; 64 Escuela de Psicología, Universidad Católica del Norte, Antofagasta, Chile; 65 Department of Psychology, University of Oslo, Oslo, Norway; 66 CIS – Centro de Investigação e Intervenção Social (CIS-ISCTE), ISCTE - Instituto Universitário de Lisboa, Lisboa, Portugal; 67 Center for Sociocultural Research, HSE University, Moscow, Russia; 68 Psychological Medicine, IoPPN, King’s College London, London, United Kingdom; 69 Department of Psychology, TOBB University of Economics and Technology, Ankara, Turkey; 70 Department of Psychology, University of Georgia, Tbilisi, Georgia; 71 School of Creative Arts Therapies, University of Haifa, Haifa, Israel; 72 Psychology Department, Effat university, Jeddah, Saudi Arabia; 73 School of professional Psychology, Bahria University, Islamabad, Pakistan; 74 Department of Psychology, National and Kapodistrian University of Athens, Athens, Greece; 75 Department of Cognitive Science and Psychology, New Bulgarian University, Sofia, Bulgaria; 76 Institute of Psychology, Vilnius University, Vilnius, Lithuania; 77 Department of Psychology, Ivane Javakhishvili Tbilisi State Univeristy, Tbilisi, Georgia; 78 Department of Sociology and Social Work, National University of Mongolia, Ulaanbaatar, Mongolia; 79 Independent Researcher, Independent Researcher, Islamabad, Pakistan; 80 Département des Sciences de l’éducation, Ecole Supérieure de l’éducation et de la Formation, Université Ibn Tofail, Kenitra, Maroc; 81 Department of Psychology, Northeast Normal University, Changchun, China; 82 Department of Pedagogy and Psychology, Kuban State University, Krasnodar, Russia; 83 Department of Psychology, Senshu University, Kawasaki, Japan; 84 Faculty of Arts and Science, Kyushu University, Fukuoka, Japan; 85 Graduate School of the Humanities, Senshu University, Kawasaki, Japan; 86 Department of Psychology, Ivan Franko National University of Lviv, Lviv, Ukraine; 87 Department of Psychology, Izmir University of Economics, Izmir, Turkey; 88 Humanities Department, Icam School of Engineering, Toulouse campus, Toulouse, France; 89 Department of Psychology, Ss. Cyril and Methodius University in Skopje, Skopje, North Macedonia; 90 Departamento de Psiquiatría y Salud Mental, Universidad de Concepción, Concepción, Chile; 91 Department of Psychology, Sapienza University, Rome, Italy; 92 Department of Psychology, Erciyes University, Kayseri, Turkey; 93 Department of Psychology, Izmir Bakırçay University, Izmir, Turkey; 94 Department of Strategy, University of Agder, Kristiansand, Norway; 95 Department of Child and Family Studies, Yonsei University, Seoul, Republic of Korea; 96 Institute of Lifelong Learning, Mendel University in Brno, Brno, Czech Republic; 97 Institute of Psychology, ELTE Eötvös Loránd University, Budapest, Hungary; 98 Department of Cognitive and Evolutionary Psychology, University of Pécs, Pécs, Hungary; 99 Department of Management, University of Agder, Kristiansand, Norway; 100 Department of Psychology, University of Pavol Jozef Šafárik in Košice, Košice, Slovakia; 101 CLLE, Université de Toulouse, Toulouse, France; 102 Department of Psychology, University of Limerick, Limerick, Ireland; 103 Laboratoire de Psychologie Sociale, Université Paris Cité, Boulogne-billancourt, France; 104 School of Psychological Sciences, University of Melbourne, Melbourne, Australia; 105 Department of Psychology, Faculty of Philosophy, University of Novi Sad, Novi Sad, Serbia; 106 Instituto de Fundamentos y Métodos en Psicología, Facultad de Psicología, Universidad de la República, Montevideo, Uruguay; 107 Department of Psychiatry, School of Medicine, Atılım University, Ankara, Turkey; 108 Louvain School of Management, Université Catholique de Louvain, Louvain La Neuve, Belgium; 109 Department of psychology, Universidad del Norte, Barranquilla, Colombia; 110 MIT Sloan School of Management, Massachusetts Institute of Technology, Cambridge, USA; 111 Department of Psychology, University of Maribor, Maribor, Slovenia; 112 School of Liberal Arts, M. Narikbayev KAZGUU University, Astana, Kazakhstan; 113 Department of Life Sciences, University of Trieste, Trieste, Italy; 114 Department of Psychology, Saint Petersburg State University, Saint Petersburg, Russia; 115 Department of Psychology, Educational Science and Human Movement, University of Palermo, Palermo, Italy; 116 Department of Psychology, University of Konstanz, Konstanz, Germany; 117 Department of Psychology, Università Cattolica del Sacro Cuore, Milano, Italy; 118 Chair of Marketing, University of Zurich, Zurich, Switzerland; 119 Bern University of Applied Sciences, Bern, Switzerland; 120 Department of Psychology, Federal Fluminense University, Campos Dos Goytacazes, Brazil; 121 Department of Psychometric - Institute of Psychology, Federal University of Rio de Janeiro, Rio de Janeiro, Brazil; 122 Department of Psychology, Sapienza University of Rome, Rome, Italy; 123 Department of Psychology, Ankara Medipol University, Ankara, Turkey; 124 Department of Experimental Psychology, Institute of Psychology, University of Sao Paulo, São Paulo, Brazil; 125 Department of Psychology, Baskent University, Ankara, Turkey; 126 Department of Psychology, University of Zagreb, Zagreb, Croatia; 127 Department of Organization, Management, and Human Resource, ESSCA School of Management, Bouloge-Billancourt, France; 128 Psychology, Pamukkale University, Denizli, Turkey; 129 Department of Psychology, University of Uberaba, Uberaba, Brazil; 130 Department of Psychology, Franklin and Marshall College, Lancaster, USA; 131 Department of Neurosciences and Developmental Biology, University of Vienna, Vienna, Austria; 132 Department of Psychology, University of Sarajevo, Sarajevo, Bosnia and Herzegovina; 133 Department of Psychology, Neapolis University Pafos, Paphos, Cyprus; 134 Department of Psychology, University of Milano-Bicocca, Milano, Italy; 135 Faculty of Life Sciences, University of Vienna, Vienna, Austria; 136 Cognitive Science Hub, University of Vienna, Vienna, Austria; 137 Urban Human, Vienna, Austria; 138 Department of Education, Literatures, Intercultural Studies, Languages and Psychology, University of Florence, Florence, Italy; 139 Department of Dermatology & Andrology, Alexandria University, Alexandria, Egypt; 140 CIS-Iscte, Instituto Universitário de Lisboa (Iscte-IUL), Lisbon, Portugal; 141 Department of Psychology, Sakarya University, Sakarya, Turkey; 142 HEI-Lab, Lusófona University, Porto, Portugal; 143 Crean College of Health and Behavioral Sciences, Chapman University, Orange, USA; 144 Department of Pedagogy, University of Wrocław, Wrocław, Poland; 145 Department of Psychology, University of Santo Tomas, Manila, Philippines; 146 Research Center for Social Sciences and Education, University of Santo Tomas, Manila, Philippines; 147 Carrera de Psicología, Universidad Politécnica Salesiana (UPS), Quito, Ecuador; 148 Faculty of Medicine of Tunis, Tunis El Manar University, Tunis, Tunisia; 149 Department of Psychiatry Ibn Omrane, Razi Hospital, Manouba, Tunisia; 150 Department of Developmental and Educational Psychology, Universidad Autónoma de Madrid, Madrid, Spain; 151 Faculty of Teacher Training and Education, Autonomous University of Madrid, Madrid, Spain; 152 Centro Interdisciplinario de Investigaciones en Psicología Matemática y Experimental, CONICET, Buenos Aires, Argentina; 153 Department of Psychology, SWPS University, Warsaw, Poland; 154 Department of Psychology and Psychotherapy, Witten/Herdecke University, Witten, Germany; 155 School of Psychological Sciences, National Autonomous University of Honduras, Tegucigalpa, Honduras; 156 Department of Psychology, Alex Ekwueme Federal University, Ndufu-alike, Nigeria; 157 School of Psychology, Wenzhou-Kean University, Wenzhou, China; 158 Administracja i Bezpieczeństwo Wewnętrzne, Uniwersytet WSB Merito Opole, Opole, Polska; 159 Faculty of Humanities and Education, Universidad Nacional Pedro Henríquez Ureña, Santo Domingo, Dominican Republic; 160 Department of Psychology, Université de Toulouse, Toulouse, France; 161 Department of Psychology, University of Crete, Rethymno, Greece; 162 The Department of Theoretical and Practical Psychology, Информация Kazakh National Women’s Teacher Training University, Almaty, Kazakhstan; 163 Department of Psychology, Michigan State University, East Lansing, MI USA; 164 Department of Mental Health, Gulu University, Kampala, Uganda; 165 Departement of Humanities, University of Urbino Carlo Bo, Urbino, Italy; 166 Department of Psychology and Behavioural Sciences, Aarhus University, Aarhus, Denmark; 167 Escuela de Pscicología, Pontificia Universidad del Ecuador- Ambato, Ambato, Ecuador; 168 Department of Education, Women University Mardan KP, Pakistan, Mardan, KP Pakistan; 169 Department of Psychology, Saint Mary’s University, Halifax, Canada; 170 Department of Psychology, University of the Philippines Diliman, Quezon City, Philippines; 171 School of Education, Tyumen State University, Tyumen, Russia; 172 Faculty of Earth Sciences and Spatial Management, Nicolaus Copernicus University, Toruń, Poland; 173 Faculty of Psychology, Universidad Cooperativa de Colombia, Medellín, Colombia; 174 Department of Political Sciences and Political Technologies, al-Farabi Kazakh National University, Almaty, Kazakhstan; 175 Department of Psychology, University of Colorado, Colorado Springs, USA; 176 Center for Cognitive Archaeology, University of Colorado, Colorado Springs, USA; 177 Institute for Advanced Study in Toulouse (IAST), Toulouse, France; 178 Lyda Hill Institute or Human Resilience, University of Colorado, Colorado Springs, USA; 179 Psychology School, Universidad Católica Andrés Bello, Caracas, Venezuela; 180 Departament of Behavioral Science and Technology, Universidad Simón Bolívar, Caracas, Venezuela; 181 School of Archaeology and Anthropology, The Australian National University, Canberra, Australia; 182 Department of Psychology, Bursa Technical University, Bursa, Turkey; 183 School of Economics, University of Asia and the Pacific, Pasig, Philippines; 184 Department of Sociology & Anthropology, University of Nigeria, Nsukka, Nigeria; 185 Kultivating and Healing, KAHLE Journey, Kingston, Jamaica; 186 School of Psychology and Vision Sciences, University of Leicester, Leicester, United Kingdom; 187 Institute of Psychology, University College of Professional Education, Wrocław, Poland; 188 Departamento de Ciências Médicas, Universidade da Beira Interior, Covilhã, Portugal

**Keywords:** Human behaviour, Human behaviour

## Abstract

Psychological studies on close relationships have often overlooked cultural diversity, dynamic processes, and potentially universal principles that shape intimate partnerships. To address the limited generalizability of previous research and advance our understanding of romantic love experiences, mate preferences, and physical attractiveness, we conducted a large-scale cross-cultural survey study on these topics. A total of 404 researchers collected data in 45 languages from April to August 2021, involving 117,293 participants from 175 countries. Aside from standard demographic questions, the survey included valuable information on variables relevant to romantic relationships: intimate, passionate, and committed love within romantic relationships, physical-attractiveness enhancing behaviors, gender equality endorsement, collectivistic attitudes, personal history of pathogenic diseases, relationship quality, jealousy, personal involvement in sexual and/or emotional infidelity, relational mobility, mate preferences, and acceptance of sugar relationships. The resulting dataset provides a rich resource for investigating patterns within, and associations across, a broad range of variables relevant to romantic relationships, with extensive opportunities to analyze individual experiences worldwide.

## Background & Summary

Studies from the early 21^st^ century have revealed a significant bias in social science research, with most studies conducted in WEIRD (Western, Educated, Industrialized, Rich, and Democratic) countries^1–3^. Research on close relationships is no exception. To illustrate, Klein *et al*.^4^ analyzed five high-impact journals dedicated to sexuality and found that a substantial majority of studies (ranging from 68% to 88%) drew samples from WEIRD populations. Bode and Kowal^5^ reported similar results in their review of the biological underpinnings of passionate love: Only 11 out of 42 (26%) studies were conducted outside of WEIRD countries.

**Fig. 1:**
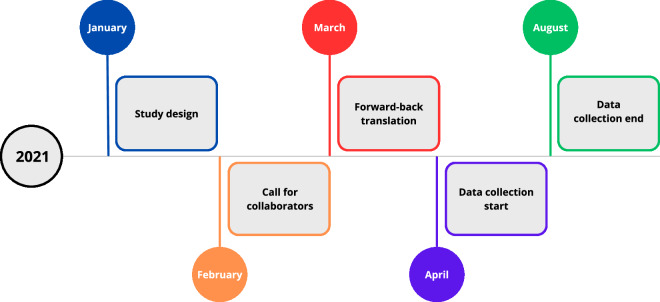
A schematic overview of the study flow.

Fortunately, the situation is gradually improving^6,7^, with more researchers emphasizing the need to ‘go beyond’ WEIRD samples. Indeed, the number of studies in non-WEIRD countries published in high-impact journals is on the rise^6^, as is the number of cross-cultural studies covering multiple countries from various continents^8^. To contribute to this growing body of research, moving beyond WEIRD samples, and to improve the generalizability of research on close relationships, we conducted a large-scale cross-cultural study involving 404 researchers from 175 countries (see Fig. 1), focusing on pair bonds, their dynamics, and cultural and environmental factors that may potentially relate to such relationships.

Pair bonds are commonly defined as dyadic attachments between two reproductive partners which last more than one reproductive cycle^9^. Although pair bonding is considered one of the most crucial aspects of reproductive behavior for some species, it is also exceptionally rare across some taxa. While 90% of bird species exhibit features of monogamous pair bonding, monogamy is relatively rare in mammals (3% to 9%)^10,11^. Among great apes, only humans engage in pair-bonding^12^. Consequently, human pair-bonding has received scholarly attention across a variety of theoretical perspectives. For instance, evolutionary approaches highlight the importance of finding a suitable mate likely to invest in offspring^13^. Developmental psychology posits that establishing an intimate romantic bond with a long-term partner is one of the key stages in human development^14^. Sociocultural theory underscores the societal importance of this phenomenon, suggesting that pair bonding is influenced by cultural norms that can shape the initiation, progression, and dynamics of romantic relationships^15^.

Given the profound impact of romantic relationships on individuals’ lives, extensive research efforts have been dedicated to exploring the antecedents of forming committed partnerships. Many empirical studies have focused on mate preferences, identifying the traits that make individuals highly attractive in the mating market. The underlying logic is that certain characteristics—such as physical attractiveness, intelligence, honesty, health, and kindness—may enhance one’s success in attracting potential partners^13,16^. Some have even suggested that a person’s desirability on the mating market may be estimated mathematically^17^. Such statistical models might be tested on large datasets, for which the present dataset could be well suited.

Yet, possessing desirable traits does not guarantee that a person will be chosen as a life partner. The solution to this enigma might lie in ‘a matter of heart’^18^ or, expressed in more scientific terms, in romantic love. Mate preferences and sexual drive toward particular individuals might stem from or lead to feelings of romantic love^19^. According to the commitment device hypothesis, romantic love evolved to foster commitment between partners, thereby enhancing their reproductive success^20^. Previous research has provided evidence that although romantic love is a nearly universal human experience^21–23^, there is substantial cultural and environmental variation in love experiences that reflect, for example, evolutionary legacy, modernization, collectivism, and average annual temperatures^24^ (for a review, see^25^). Such hypotheses might be further explored with the present cross-cultural dataset containing information on love experiences with measures that have already been cross-culturally validated^22,26^. Furthermore, once a romantic relationship is formed, numerous other affective phenomena emerge, including feelings of jealousy, relationship satisfaction, and commitment—all of which were also measured in the present dataset. In past research, these phenomena have been examined in isolation and primarily studied in a limited number of countries (for reviews, see^27,28^).

Here, to broaden the scope of close relationship research, we address the existing gap and offer a large-scale cross-cultural dataset. It consists of a comprehensive collection of variables on demographics, intimacy, passion, and commitment within romantic relationships, physical attractiveness enhancing behaviors, gender equality endorsement, collectivistic attitudes, personal history of pathogenic diseases, relationship quality, jealousy, infidelity, relational mobility, mate preferences, current mate ratings, self-ratings, and acceptance of sugar relationships. Furthermore, because our survey was translated into 45 languages, it provides a basis for validating various linguistic versions of the scales used (e.g.,^22,26^).

## Methods

When describing the following section, we used articles presenting social sciences data published in Scientific Data as blueprints^29–31^.

### Ethical considerations

The protocol for this study was approved by the Institutional Review Board (IRB) at the Institute of Psychology, University of Wrocław (number IPE0022). Data collection was conducted by team members in accordance with the ethical guidelines established by their respective IRBs, following either the Principal Investigator’s IRB approval or the ethical clearances obtained from their local IRBs. Furthermore, all participants provided informed consent prior to their involvement in the study. Specifically, they confirmed that they were over 18 years old and acknowledged that their data, anonymized and stripped of any identifiable information, would be analyzed and disseminated in scientific reports and papers. Anonymity and confidentiality were guaranteed to participants as well as the voluntary nature of their participation.

### Survey

**Fig. 2:**
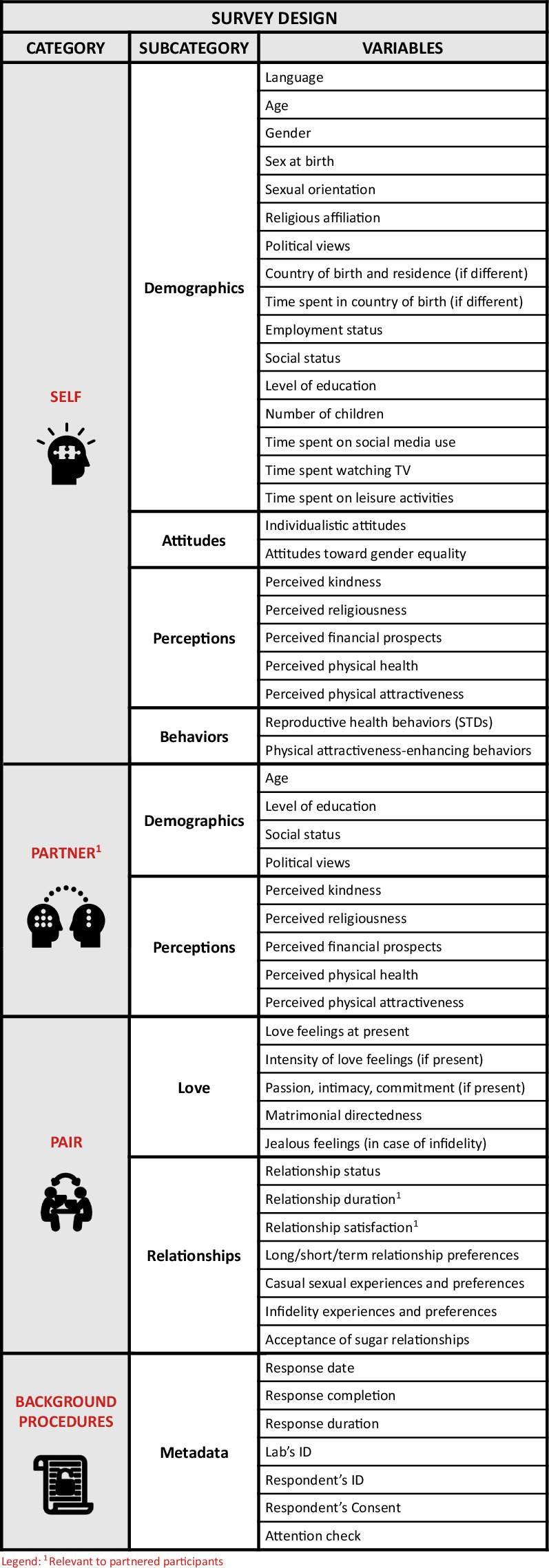
A visual overview of the study’s content (for detailed list of all variables, see Codebook).

The English version of the survey, along with all 45 translated linguistic versions, can be accessed on the Open Science Framework^32^. It contains the following sections (for the visual overview of the survey’s content, see Fig. 2):

1. **Demographics**: Gender, sex at birth, age, country of birth and residence (if different), time spent in country of birth, relationship status, number of children, religious affiliation, sexual orientation, employment status, average daily time spent on social media, on TV, and time spent on leisure activities.
2. **Romantic love**: For partnered individuals: the Triangular Love Scale (TLS-15^22,33^), relationship length. For all individuals: Kephart’s^18^ question on the importance of romantic love^20^, being in love with anyone and the strength of these love feelings.
3. **Gender equality**: A subscale of the Gender Equitable Men Scale^34^.
4. **Collectivistic attitudes:** A subscale of the Collectivism Scale^35^.
5. **Personal Pathogen History:** Pathogen Prevalence Index^36^.
6. **Physical Attractiveness-Enhancing Behaviors Scale**: Importance and time spent on eight types of physical-attractiveness enhancing behaviors^26^.
7. **Long-term relationship preferences:** Six items adapted from the MPQ15^17^.
8. **Short-term relationship preferences:** Six items adapted from the MPQ15 ^17^.
9. **Preference Importance Measure:** 30 points allocated across six traits, including health, kindness, physical attractiveness, religiousness, financial prospects, and correct age, inspired by the budget allocation method used in Li *et al*.^37^ and Conroy-Beam *et al*.^17^.
10. **Self-ratings:** Six items adapted from the MPQ15^17^.
11. **Mate ratings:** Six items adapted from the MPQ15^17^.
12. **Relationship satisfaction**: Six items from the 18-item Perceived Relationship Quality Components (PRQC^38^).
13. **Jealousy Scale**: Two items adapted from Buss *et al*.^39^.
14. **Sociosexual Attitudes Scale**: Three items from the revised Sociosexual Orientation Inventory (SOI-R^40^).
15. **Infidelity Scale**: Two items assessing the perceived morality of sexual and emotional infidelity, developed based on findings from Carpenter’s^41^ meta-analysis.
16. **Acceptance of Sugar Relationships**: Developed for younger companion providers (ASR-YWMS) and older resource providers (ASR-OMWS)^42^.

A detailed list of all items with their response ranges and codes can be found in the Codebook file titled “Codebook.xlsx” on the Open Science Framework, OSF^32^. The survey also included the Dance Perceptions Scale. However, this portion of the data is not included in the current dataset, as it is reserved for a forthcoming publication within the scope of a larger, long-term project on dance perceptions. These data will be made available upon the release of the final paper.

### Participants

In total, we collected 119,781 responses. Excluding data from those who did not consent to participate in the study (*n* = 639), who previewed (*n* = 4) or tested the survey (*n* = 549) but did not complete it, who mistakenly doubled their submission (*n* = 21), who were recruited by one team member who collected data before asking their local IRB for approval, which violated the local IRB’s rules, and thus was asked to withdraw the data (*n* = 244), and who failed the attention check (*n = *1031) resulted in a final dataset of 117,293 participants from 175 countries. Out of these, 71,361 participants (61%) from 158 countries completed the whole survey, whereas 86,966 (74%) from 165 countries completed at least half of the survey.

**Fig. 3:**
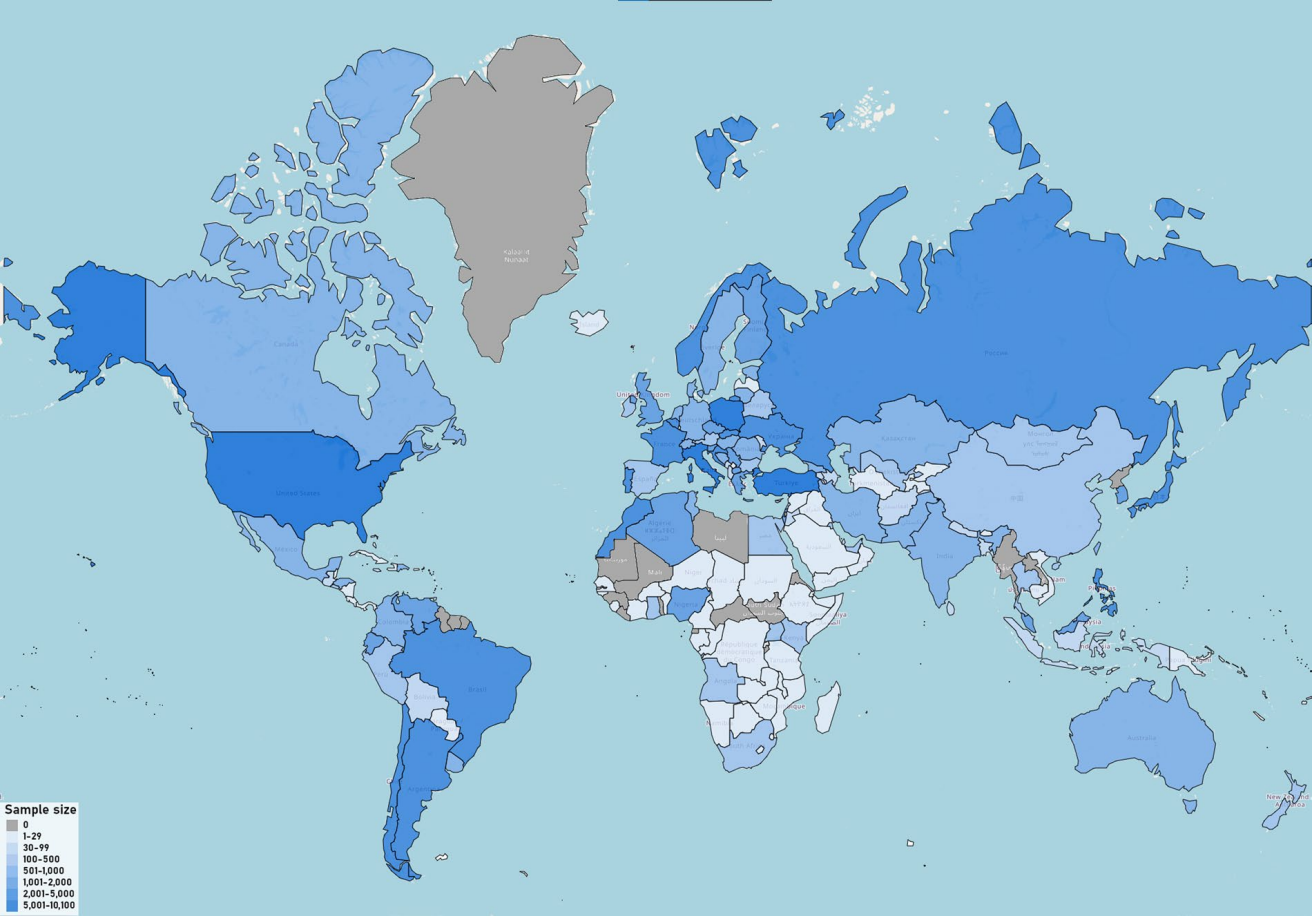
A world map visualizing the number of participants across countries, with the color scale representing the sample size (the darker, the larger). *Note*. Gray areas represent countries not covered by the data.

Basic demographic characteristics of participants from the final dataset are presented in Table 1, whereas demographic characteristics across countries with at least 30 participants (*k* = 97) are given in the “Demographics across countries.xlsx” file on the OSF^32^. Figure 3 shows where the data were collected, colored according to the sample size.

**Table 1:** Basic demographic characteristics of the final sample (*N* = 117,293).

Variable		N/Mean	%/SD
Gender			
	Women	71327	60.80%
	Men	34864	29.70%
	Non-binary	1089	0.90%
	Prefer not to say	486	0.40%
	N/A	9527	8.10%
Age		30.35	12.55
Number of children		0.50	0.97
Education			
	No formal education	275	0.20%
	Primary school only	474	0.40%
	Primary school through Secondary school	38763	33.00%
	Primary school through High school or Technical college	19535	16.70%
	Primary school through Bachelor's degree	18005	15.40%
	Primary school through Master's degree	5473	4.70%
	Primary school through PhD, MD, JD, or other advanced degree	5216	4.40%
	N/A	29552	25.20%
Religious affiliation			
	Buddhism	2362	2%
	Christianity	41486	35.40%
	Hinduism	1033	0.90%
	Islam	16820	14.30%
	Judaism	1331	1.10%
	None	40253	34.30%
	Other	4106	3.50%
	N/A	9902	8.40%
Sexual orientation			
	Asexual	1110	0.90%
	Bisexual	7895	6.70%
	Gay	2156	1.80%
	Heterosexual	87043	74.20%
	Lesbian	1455	1.20%
	Other	1244	1.10%
	Pansexual	1863	1.60%
	Prefer not to say	4229	3.60%
	N/A	10298	8.80%

### Translations

The English version of the survey embedded in the HTML codes was pasted into 45 separate Google Spreadsheet files. Each of the 45 translation teams, consisting of bilingual collaborators, received a separate Google Spreadsheet file that consisted of four sheets. On the first sheet, there were instructions on how to perform the forward-back translation^43^. The second sheet was intended for the forward-translation of the survey from English into a local language. The third sheet was intended for back-translation from the same local language into English. The final sheet was intended to prepare the final version of the survey in the local language. One or more native speakers performed the forward-translation, then other(s) performed the back-translation, and, finally, both forward- and back-translation teams discussed the differences, agreed on their resolution, and prepared the final linguistic version of the survey. Detailed instructions, along with a short video explaining the translation task, are presented in the “Instructions for translating teams.docx” file on the OSF^32^. All linguistic versions of the survey can be accessed in the “Translated survey - all languages.xlsx” file and “Translation Farsi.doc” file on the OSF^32^.

### Procedure

After the translation process was completed, the study for that given language was launched, starting with English and Polish on April 8, 2021, to validate the Qualtrics survey and data protocols. Data collection was conducted over a span of five months, from April to August 2021. Data were collected primarily online via the Qualtrics website or related online platforms, except for two countries (i.e., Algeria and Morocco), where potential participants could not access the Qualtrics website for technical reasons. Therefore, the team members collected data from these two countries in person using a paper-and-pencil method. Moreover, due to difficulties accessing the Qualtrics website in Iran, we recreated the survey and collected data using Google Forms. Lastly, one Russian Collaborator collected data via the Toloka website (a crowdsourcing platform popular in Russia, similar to Prolific or Mechanical Turk). Collaborators strived to collect data from as diverse samples as possible, including inviting participants of various ages, genders, from various regions (including rural and urban areas), from the community and university samples, and so forth. While answering the survey, participants were also encouraged to share the link to the survey on their social media platforms with an already prepared invitation text (see the “Invitation text.docx” file on the OSF^32^). Approximately 6% of the data were collected using outsourcing platforms (e.g., Prolific, MTurk, Toloka).

### Data cleaning

Most collaborators collected data via personalized Qualtrics links to the general survey (with a few exceptions, described in the Procedure paragraph). Moreover, due to certain collaborators’ requests, connected with, for instance, adding personalized information within the survey, redirections to other websites, the need for better monitoring of data inflow, or collecting more information to grant course credits to students who helped with data collection, seven labs recruited participants through separate Qualtrics branches. All these datasets were merged into the final, ready-to-use dataset.

Data coming from outside of the general survey link were prone to minor coding mistakes. For instance, manually prepared datasets from Algeria and Morocco contained typos, such as doubled scores (e.g., a response of “44” on a 1–5 scale range). All these errors are addressed in the final dataset, the details for which are described in detail in the R script available on the OSF (see below).

## Data Records

All materials associated with this large-scale, cross-cultural project can be found on the project’s repository (comprising four folders) on the OSF^32^. The folder entitled “Dataset” contains the final, ready-to-use dataset (named “Final_dataset.csv”) and the Codebook of all variables with their response ranges (named “Codebook.xlsx”). The folder “R code” consists of a .txt file (named “R script.txt”) that was run to merge and clean raw datasets. Raw datasets are not shared because they may contain personal information about participants and collaborators (e.g., email addresses, student identification numbers, and detailed names of university groups and courses). The full anonymization code is available in the file “R Script.txt.” The folder “Survey” contains the .docx files “Instructions for translating teams.docx” and “Invitation text.docx”, which aimed to encourage participants to share the link to the survey with their friends, families, and on social media. The Survey folder also contains the .pdf file with the English version of the survey (named “Large_scale_project_English_with_codes.pdf”), the “README.txt” file which reminds users about any incongruence between the coding from the .pdf version of the survey and final coding in the dataset (as explained in the “Usage Notes” section), as well as .xlsx and .docx files with all translated versions of the survey (named “Translated survey - all languages.xlsx” and “Translation Farsi.docx”). The folder “R code” contains a .txt file (named “R code large-scale study.txt”) with R code that was used to prepare the final dataset. Finally, the “Data description” folder contains the .xlsx file (named “Demographics across countries.xlsx”) with demographic characteristics of participants for countries with at least 30 participants. It also contains the “Reliabilities across countries.xlsx” file containing information on the reliability of the multi-item scales across these countries, and the “Means across countries.xlsx” file with the means of the scales across these countries.

## Technical Validation

For technical validation, we examined the data quality (e.g., Cronbach’s alpha scores, correlations) from 97 countries with at least 30 participants. Overall, 70 of these 97 countries (72%) had more than 200 participants, whereas 32 of them had more than 1,000 participants. The average age in this sample was similar to that of the whole sample, that is, 30.35 (*SD* = 12.54), but varied across the countries, ranging from 21.48 (*SD* = 5.06) in Thailand to 47.32 (*SD* = 16.86) in Argentina. The proportion of women in this sample was 66.30%, and again, this varied across countries, ranging from 5.4% in Ghana to 84% in Greece. The proportion of individuals who attained a tertiary level of education (i.e., Bachelor’s degree or higher) was 58%, and also differed across countries, ranging from 6.1% in Ghana to 81.4% in Kenya. Although we cannot determine the representativeness of the included country populations, we believe that the data are still valuable in examining important research questions across a range of cultures varying widely in their norms surrounding relationships, sexual behavior, sexuality, and mate selection.

Despite variation in demographic variables across countries, the internal consistency of the scales ranged from good to excellent. For instance, Cronbach’s alpha for the TLS-15 = 0.94, Intimacy = 0.90, Passion = 0.87, Commitment = 0.89^22^, gender equality = 0.85^34^, collectivism = 0.76^35^, Perceived Relationship Quality Components (PRQC) = 0.93, relationship satisfaction = 0.94, relationship commitment = 0.89^38^, Acceptance of Sugar Relationships = 0.95, receiving subscale = 0.93, giving subscale = 0.93^42^. The “Reliabilities across countries.xlsx” file (accessible on the OSF^32^) presents Cronbach’s alphas for the scales across countries, with at least 30 participants answering the given scale. Basic descriptive statistics for these scales, including means and standard deviations, are presented in the “Means across countries.xlsx” file (accessible on the OSF^32^).

For further validation of the data, we investigated correlations across variables that should correlate and those for which there are no expected associations. For example, we predicted high positive Pearson correlations between age and relationship length and age and the number of children^40^. Indeed, that is what we observed (*r =* 0.74 and *r* = 0.62, respectively). Similarly, we expected high positive correlations between subscales of the Triangular Love Scale (TLS-15^22^) and relationship satisfaction^38^: Intimacy *r* = 0.70, Passion *r =* 0.63, Commitment *r =* 0.63. In line with previous research^44^, we also observed a high correlation between one’s own and partner’s age (*r =* 0.87). Conversely, we did not expect to see any significant links between subscales of love and time spent on social media, and, indeed, such correlations were marginal in effect size (Intimacy *r =* 0.04, Passion *r =* 0.07, Commitment *r =* 0.002).

## Usage Notes

The data are freely available, cleaned, and ready for analyses. We recommend that interested researchers first consult the codebook (“Codebook.xslx”) before using the final version of the dataset (available on OSF^32^). The codebook presents all variables in the final dataset along with a brief explanation of both their scoring and what they represent. This is especially important because the original coding of some of the variables described in the .pdf version of the survey was recoded to be more intuitive. For example, there is a demographic question regarding the participant’s social class in the .pdf version of the survey, originally coded so that higher values represent lower social classes. In the final dataset, however, this item was reversed so that higher values represent higher social classes. In general, the naming of the variables follows the underlying logic so that higher scores represent “more” of the given psychological construct. Therefore, for instance, the “Gender_equality” items denote the participant’s agreement with more gender-equal views. Variables can be used individually or with the calculated average scores. To identify individuals from a specific country, the variables “Country_live” or “Country_raised” should be used, depending on whether researchers wish to use participants’ country of residence, the country in which they were raised, or both.

Additionally, interested researchers should be cautious about using the mate budget allocation task, in which participants had to distribute 30 points across six potential traits (health, kindness, physical attractivity, religiousness, financial prospects, correct age) in a romantic partner. When collecting data with the paper-and-pencil method in Algeria and Morocco, there was no validation of the total sum of allocated points. Consequently, the sum of allocated points exceeds 30 in almost 100 Algerian participants.

## Supplementary information

Supplementary Material

## Data Availability

The R code for cleaning is available on the OSF^32^.
