# Supplementary material for: Cross-cultural data on romantic love and mate preferences from 117,293 participants across 175 countries
Source: Sci Data. 2025 Jul 1;12:1103. doi: 10.1038/s41597-025-05365-2 (PMC12219412; doi:10.1038/s41597-025-05365-2)
Supplement: Supplementary file 1 — Supplementary Material [file 41597_2025_5365_MOESM1_ESM.docx]

**Supplementary Material**

**Figure S1**

Authors’ contribution.

| **Researcher** | **Conceptualization** | **Data curation** | **Formal analysis** | **Funding acqusition** | **Investigation** | **Methodology** | **Project administration** | **Resources** | **Software** | **Supervision** | **Validation** | **Visualization** | **Writing (original draft)** | **Writing (review & editing)** |
| --- | --- | --- | --- | --- | --- | --- | --- | --- | --- | --- | --- | --- | --- | --- |
| Marta Kowal |  |  |  |  |  |  |  |  |  |  |  |  |  |  |
| Piotr Sorokowski |  |  |  |  |  |  |  |  |  |  |  |  |  |  |
| Biljana Gjoneska |  |  |  |  |  |  |  |  |  |  |  |  |  |  |
| Katarzyna Pisanski |  |  |  |  |  |  |  |  |  |  |  |  |  |  |
| Gerit Pfuhl |  |  |  |  |  |  |  |  |  |  |  |  |  |  |
| Leonardo Aguilar |  |  |  |  |  |  |  |  |  |  |  |  |  |  |
| Steve M.J. Janssen |  |  |  |  |  |  |  |  |  |  |  |  |  |  |
| Benjamin Gelbart |  |  |  |  |  |  |  |  |  |  |  |  |  |  |
| Patrícia Arriaga |  |  |  |  |  |  |  |  |  |  |  |  |  |  |
| Jan Antfolk |  |  |  |  |  |  |  |  |  |  |  |  |  |  |
| Katarina Zvončáková |  |  |  |  |  |  |  |  |  |  |  |  |  |  |
| Linda H. Lidborg |  |  |  |  |  |  |  |  |  |  |  |  |  |  |
| Jorge Contreras-Garduño |  |  |  |  |  |  |  |  |  |  |  |  |  |  |
| Mikhail V. Kozlov |  |  |  |  |  |  |  |  |  |  |  |  |  |  |
| Taciano L. Milfont |  |  |  |  |  |  |  |  |  |  |  |  |  |  |
| Marco Varella |  |  |  |  |  |  |  |  |  |  |  |  |  |  |
| Valerija Križanić |  |  |  |  |  |  |  |  |  |  |  |  |  |  |
| Mahmoud Boussena |  |  |  |  |  |  |  |  |  |  |  |  |  |  |
| Tina Kavčič |  |  |  |  |  |  |  |  |  |  |  |  |  |  |
| Diana R.D. Silva |  |  |  |  |  |  |  |  |  |  |  |  |  |  |
| Brahim Hamdaoui |  |  |  |  |  |  |  |  |  |  |  |  |  |  |
| Fatima Zahra Sahli |  |  |  |  |  |  |  |  |  |  |  |  |  |  |
| Karlijn Massar |  |  |  |  |  |  |  |  |  |  |  |  |  |  |
| Eliane Deschrijver |  |  |  |  |  |  |  |  |  |  |  |  |  |  |
| Tatsunori Ishii |  |  |  |  |  |  |  |  |  |  |  |  |  |  |
| Hakan Cetinkaya |  |  |  |  |  |  |  |  |  |  |  |  |  |  |
| Oksana Senyk |  |  |  |  |  |  |  |  |  |  |  |  |  |  |
| Farida Guemaz |  |  |  |  |  |  |  |  |  |  |  |  |  |  |
| Koen Ponnet |  |  |  |  |  |  |  |  |  |  |  |  |  |  |
| Yahya Don |  |  |  |  |  |  |  |  |  |  |  |  |  |  |
| Dušana Šakan |  |  |  |  |  |  |  |  |  |  |  |  |  |  |
| Gyesook Yoo |  |  |  |  |  |  |  |  |  |  |  |  |  |  |
| Ravit Nussinson |  |  |  |  |  |  |  |  |  |  |  |  |  |  |
| Joaquín Ungaretti |  |  |  |  |  |  |  |  |  |  |  |  |  |  |
| Ali R. Can |  |  |  |  |  |  |  |  |  |  |  |  |  |  |
| Izzet Duyar |  |  |  |  |  |  |  |  |  |  |  |  |  |  |
| Jiří Čeněk |  |  |  |  |  |  |  |  |  |  |  |  |  |  |
| Joao Carneiro |  |  |  |  |  |  |  |  |  |  |  |  |  |  |
| Norbert Meskó |  |  |  |  |  |  |  |  |  |  |  |  |  |  |
| Luca Kozma |  |  |  |  |  |  |  |  |  |  |  |  |  |  |
| Ellen K. Nyhus |  |  |  |  |  |  |  |  |  |  |  |  |  |  |
| Mona Vintila |  |  |  |  |  |  |  |  |  |  |  |  |  |  |
| Oulmann Zerhouni |  |  |  |  |  |  |  |  |  |  |  |  |  |  |
| Farid Pazhoohi |  |  |  |  |  |  |  |  |  |  |  |  |  |  |
| Maja Zupančič |  |  |  |  |  |  |  |  |  |  |  |  |  |  |
| Sinem Söylemez |  |  |  |  |  |  |  |  |  |  |  |  |  |  |
| Austin H.-E. Wang |  |  |  |  |  |  |  |  |  |  |  |  |  |  |
| Marietta Papadatou-Pastou |  |  |  |  |  |  |  |  |  |  |  |  |  |  |
| Irena Pavela Banai |  |  |  |  |  |  |  |  |  |  |  |  |  |  |
| Pavol Prokop |  |  |  |  |  |  |  |  |  |  |  |  |  |  |
| Mohd Sofian B. Omar Fauzee |  |  |  |  |  |  |  |  |  |  |  |  |  |  |
| Reza Afhami |  |  |  |  |  |  |  |  |  |  |  |  |  |  |
| Jean C. Natividade |  |  |  |  |  |  |  |  |  |  |  |  |  |  |
| Roberto Baiocco |  |  |  |  |  |  |  |  |  |  |  |  |  |  |
| Mara Morelli |  |  |  |  |  |  |  |  |  |  |  |  |  |  |
| Toivo Aavik |  |  |  |  |  |  |  |  |  |  |  |  |  |  |
| Ezgi Toplu-Demirtaş |  |  |  |  |  |  |  |  |  |  |  |  |  |  |
| Singha Tulyakul |  |  |  |  |  |  |  |  |  |  |  |  |  |  |
| Anna Wlodarczyk |  |  |  |  |  |  |  |  |  |  |  |  |  |  |
| Razieh Chegeni |  |  |  |  |  |  |  |  |  |  |  |  |  |  |
| Anabela C. Santos |  |  |  |  |  |  |  |  |  |  |  |  |  |  |
| Dmitry Grigoryev |  |  |  |  |  |  |  |  |  |  |  |  |  |  |
| Dmitrii Dubrov |  |  |  |  |  |  |  |  |  |  |  |  |  |  |
| Dimitri Chubinidze |  |  |  |  |  |  |  |  |  |  |  |  |  |  |
| Gözde Ikizer |  |  |  |  |  |  |  |  |  |  |  |  |  |  |
| Nana Burduli |  |  |  |  |  |  |  |  |  |  |  |  |  |  |
| Johanna Czamanski-Cohen |  |  |  |  |  |  |  |  |  |  |  |  |  |  |
| Rizwana Amin |  |  |  |  |  |  |  |  |  |  |  |  |  |  |
| Petros Roussos |  |  |  |  |  |  |  |  |  |  |  |  |  |  |
| Evgeniya Hristova |  |  |  |  |  |  |  |  |  |  |  |  |  |  |
| Rūta Sargautytė |  |  |  |  |  |  |  |  |  |  |  |  |  |  |
| Ekaterine Pirtskhalava |  |  |  |  |  |  |  |  |  |  |  |  |  |  |
| Tenuunjargal Avirmed |  |  |  |  |  |  |  |  |  |  |  |  |  |  |
| Arooj Najmussaqib |  |  |  |  |  |  |  |  |  |  |  |  |  |  |
| Abdelilah Charyate |  |  |  |  |  |  |  |  |  |  |  |  |  |  |
| Shagufta Batool |  |  |  |  |  |  |  |  |  |  |  |  |  |  |
| Tatiana Volkodav |  |  |  |  |  |  |  |  |  |  |  |  |  |  |
| Yoshihiko Kunisato |  |  |  |  |  |  |  |  |  |  |  |  |  |  |
| Yuki Yamada |  |  |  |  |  |  |  |  |  |  |  |  |  |  |
| Asako Toyama |  |  |  |  |  |  |  |  |  |  |  |  |  |  |
| Mariia Perun |  |  |  |  |  |  |  |  |  |  |  |  |  |  |
| Seda Dural |  |  |  |  |  |  |  |  |  |  |  |  |  |  |
| Tetyana Mandzyk |  |  |  |  |  |  |  |  |  |  |  |  |  |  |
| Anna Studzinska |  |  |  |  |  |  |  |  |  |  |  |  |  |  |
| Ognen Spasovski |  |  |  |  |  |  |  |  |  |  |  |  |  |  |
| Felipe E. García |  |  |  |  |  |  |  |  |  |  |  |  |  |  |
| Caterina Grano |  |  |  |  |  |  |  |  |  |  |  |  |  |  |
| Merve Boğa |  |  |  |  |  |  |  |  |  |  |  |  |  |  |
| Mehmet Koyuncu |  |  |  |  |  |  |  |  |  |  |  |  |  |  |
| Sangeeta Singh |  |  |  |  |  |  |  |  |  |  |  |  |  |  |
| Ju Hee Park |  |  |  |  |  |  |  |  |  |  |  |  |  |  |
| Derya Atamtürk |  |  |  |  |  |  |  |  |  |  |  |  |  |  |
| Samuel Lins |  |  |  |  |  |  |  |  |  |  |  |  |  |  |
| Martin Pírko |  |  |  |  |  |  |  |  |  |  |  |  |  |  |
| David Lacko |  |  |  |  |  |  |  |  |  |  |  |  |  |  |
| Balazs Aczel |  |  |  |  |  |  |  |  |  |  |  |  |  |  |
| Ferenc Kocsor |  |  |  |  |  |  |  |  |  |  |  |  |  |  |
| Ádám Putz |  |  |  |  |  |  |  |  |  |  |  |  |  |  |
| Tobias Otterbring |  |  |  |  |  |  |  |  |  |  |  |  |  |  |
| Pavol Kačmár |  |  |  |  |  |  |  |  |  |  |  |  |  |  |
| Efisio Manunta |  |  |  |  |  |  |  |  |  |  |  |  |  |  |
| Théo Besson |  |  |  |  |  |  |  |  |  |  |  |  |  |  |
| Nasim Ghahraman Moharrampour |  |  |  |  |  |  |  |  |  |  |  |  |  |  |
| Çağlar Solak |  |  |  |  |  |  |  |  |  |  |  |  |  |  |
| Bojana M. Dinić |  |  |  |  |  |  |  |  |  |  |  |  |  |  |
| Ignacio Estevan |  |  |  |  |  |  |  |  |  |  |  |  |  |  |
| Merve Topcu Bulut |  |  |  |  |  |  |  |  |  |  |  |  |  |  |
| Nicolas Kervyn |  |  |  |  |  |  |  |  |  |  |  |  |  |  |
| Moises Mebarak |  |  |  |  |  |  |  |  |  |  |  |  |  |  |
| Jackson G. Lu |  |  |  |  |  |  |  |  |  |  |  |  |  |  |
| Nejc Plohl |  |  |  |  |  |  |  |  |  |  |  |  |  |  |
| Bojan Musil |  |  |  |  |  |  |  |  |  |  |  |  |  |  |
| Adil Samekin |  |  |  |  |  |  |  |  |  |  |  |  |  |  |
| Kirill G. Miroshnik |  |  |  |  |  |  |  |  |  |  |  |  |  |  |
| Clément Cornec |  |  |  |  |  |  |  |  |  |  |  |  |  |  |
| Isabella Giammusso |  |  |  |  |  |  |  |  |  |  |  |  |  |  |
| Ulf-Dietrich Reips |  |  |  |  |  |  |  |  |  |  |  |  |  |  |
| Maria Rosa Miccoli |  |  |  |  |  |  |  |  |  |  |  |  |  |  |
| Miriam Parise |  |  |  |  |  |  |  |  |  |  |  |  |  |  |
| Sabrina Stöckli |  |  |  |  |  |  |  |  |  |  |  |  |  |  |
| Tiago Marot |  |  |  |  |  |  |  |  |  |  |  |  |  |  |
| Sibele D. Aquino |  |  |  |  |  |  |  |  |  |  |  |  |  |  |
| Amanda Londero-Santos |  |  |  |  |  |  |  |  |  |  |  |  |  |  |
| Antonio Chirumbolo |  |  |  |  |  |  |  |  |  |  |  |  |  |  |
| Aybegum Memisoglu-Sanli |  |  |  |  |  |  |  |  |  |  |  |  |  |  |
| Jaroslava V. Valentova |  |  |  |  |  |  |  |  |  |  |  |  |  |  |
| Cemre Karaarslan |  |  |  |  |  |  |  |  |  |  |  |  |  |  |
| Ivana Hromatko |  |  |  |  |  |  |  |  |  |  |  |  |  |  |
| Kevin Sevag Kertechian |  |  |  |  |  |  |  |  |  |  |  |  |  |  |
| Ogeday Çoker |  |  |  |  |  |  |  |  |  |  |  |  |  |  |
| Matheus F. Ribeiro |  |  |  |  |  |  |  |  |  |  |  |  |  |  |
| Carlota Batres |  |  |  |  |  |  |  |  |  |  |  |  |  |  |
| Ilker Dalgar |  |  |  |  |  |  |  |  |  |  |  |  |  |  |
| Stephanie J. Eder |  |  |  |  |  |  |  |  |  |  |  |  |  |  |
| Katarina Mišetić |  |  |  |  |  |  |  |  |  |  |  |  |  |  |
| Marios Argyrides |  |  |  |  |  |  |  |  |  |  |  |  |  |  |
| Vita Mikuličiūtė |  |  |  |  |  |  |  |  |  |  |  |  |  |  |
| Silvia Mari |  |  |  |  |  |  |  |  |  |  |  |  |  |  |
| Elisabeth Oberzaucher |  |  |  |  |  |  |  |  |  |  |  |  |  |  |
| Kathrin Masuch |  |  |  |  |  |  |  |  |  |  |  |  |  |  |
| Alan D.A. Mattiassi |  |  |  |  |  |  |  |  |  |  |  |  |  |  |
| Salma S. Omar |  |  |  |  |  |  |  |  |  |  |  |  |  |  |
| Elena Piccinelli |  |  |  |  |  |  |  |  |  |  |  |  |  |  |
| Eda Ermagan Caglar |  |  |  |  |  |  |  |  |  |  |  |  |  |  |
| Diogo Lamela |  |  |  |  |  |  |  |  |  |  |  |  |  |  |
| David A. Frederick |  |  |  |  |  |  |  |  |  |  |  |  |  |  |
| Aleksander Kobylarek |  |  |  |  |  |  |  |  |  |  |  |  |  |  |
| Ma. Criselda T. Pacquing |  |  |  |  |  |  |  |  |  |  |  |  |  |  |
| Marc Eric S. Reyes |  |  |  |  |  |  |  |  |  |  |  |  |  |  |
| Marcos Zumárraga-Espinosa |  |  |  |  |  |  |  |  |  |  |  |  |  |  |
| Feten Fekih-Romdhane |  |  |  |  |  |  |  |  |  |  |  |  |  |  |
| Talía Gómez Yepes |  |  |  |  |  |  |  |  |  |  |  |  |  |  |
| Edgardo Etchezahar |  |  |  |  |  |  |  |  |  |  |  |  |  |  |
| Katarzyna Galasinska |  |  |  |  |  |  |  |  |  |  |  |  |  |  |
| Jan P. Röer |  |  |  |  |  |  |  |  |  |  |  |  |  |  |
| Ayşegül Şahin |  |  |  |  |  |  |  |  |  |  |  |  |  |  |
| Miguel Landa-Blanco |  |  |  |  |  |  |  |  |  |  |  |  |  |  |
| Izuchukwu L.G. Ndukaihe |  |  |  |  |  |  |  |  |  |  |  |  |  |  |
| Arkadiusz Urbanek |  |  |  |  |  |  |  |  |  |  |  |  |  |  |
| Chee-Seng Tan |  |  |  |  |  |  |  |  |  |  |  |  |  |  |
| Rita Castro |  |  |  |  |  |  |  |  |  |  |  |  |  |  |
| Ksenija Cunichina |  |  |  |  |  |  |  |  |  |  |  |  |  |  |
| Anna Krasnodębska |  |  |  |  |  |  |  |  |  |  |  |  |  |  |
| Daniel Conroy-Beam |  |  |  |  |  |  |  |  |  |  |  |  |  |  |
| Franciszek Ostaszewski |  |  |  |  |  |  |  |  |  |  |  |  |  |  |
| Izabela Chałatkiewicz |  |  |  |  |  |  |  |  |  |  |  |  |  |  |
| Beatriz Abad-Villaverde |  |  |  |  |  |  |  |  |  |  |  |  |  |  |
| Bastien Trémolière |  |  |  |  |  |  |  |  |  |  |  |  |  |  |
| Alexios Arvanitis |  |  |  |  |  |  |  |  |  |  |  |  |  |  |
| Gulmira T. Topanova |  |  |  |  |  |  |  |  |  |  |  |  |  |  |
| William J. Chopik |  |  |  |  |  |  |  |  |  |  |  |  |  |  |
| Grace Akello |  |  |  |  |  |  |  |  |  |  |  |  |  |  |
| Ariela F. Pagani |  |  |  |  |  |  |  |  |  |  |  |  |  |  |
| Silvia Donato |  |  |  |  |  |  |  |  |  |  |  |  |  |  |
| Peter Fedor |  |  |  |  |  |  |  |  |  |  |  |  |  |  |
| Tomasz Frackowiak |  |  |  |  |  |  |  |  |  |  |  |  |  |  |
| Simon Ozer |  |  |  |  |  |  |  |  |  |  |  |  |  |  |
| Marlon Mayorga-Lascano |  |  |  |  |  |  |  |  |  |  |  |  |  |  |
| Farah Khan |  |  |  |  |  |  |  |  |  |  |  |  |  |  |
| Maryanne L. Fisher |  |  |  |  |  |  |  |  |  |  |  |  |  |  |
| Princess Lovella G. Maturan |  |  |  |  |  |  |  |  |  |  |  |  |  |  |
| Tatiana Semenovskikh |  |  |  |  |  |  |  |  |  |  |  |  |  |  |
| Sanjana Dutt |  |  |  |  |  |  |  |  |  |  |  |  |  |  |
| William Tamayo-Agudelo |  |  |  |  |  |  |  |  |  |  |  |  |  |  |
| Gulnara Ismukhanova |  |  |  |  |  |  |  |  |  |  |  |  |  |  |
| Laith Al-Shawaf |  |  |  |  |  |  |  |  |  |  |  |  |  |  |
| Luisa Angelucci |  |  |  |  |  |  |  |  |  |  |  |  |  |  |
| Adam Bode |  |  |  |  |  |  |  |  |  |  |  |  |  |  |
| Sercan Balım |  |  |  |  |  |  |  |  |  |  |  |  |  |  |
| Jovi C. Dacanay |  |  |  |  |  |  |  |  |  |  |  |  |  |  |
| Chiemezie S. Atama |  |  |  |  |  |  |  |  |  |  |  |  |  |  |
| Kai A.D. Morgan Campbell |  |  |  |  |  |  |  |  |  |  |  |  |  |  |
| Tchilissila A. Simões |  |  |  |  |  |  |  |  |  |  |  |  |  |  |
| Barış Özener |  |  |  |  |  |  |  |  |  |  |  |  |  |  |
| Paula Błauciak |  |  |  |  |  |  |  |  |  |  |  |  |  |  |
| Filipe Prazeres |  |  |  |  |  |  |  |  |  |  |  |  |  |  |
